# Hierarchical Fusion Network with Enhanced Knowledge and Contrastive Learning for Multimodal Aspect-Based Sentiment Analysis on Social Media

**DOI:** 10.3390/s23177330

**Published:** 2023-08-22

**Authors:** Xiaoran Hu, Masayuki Yamamura

**Affiliations:** Department of Computer Science, School of Computing, Tokyo Institute of Technology, 4259 Nagatsuta, Midori-ku, Yokohama-shi 226-8502, Japan

**Keywords:** aspect-based sentiment analysis, social data fusion, signal processing, knowledge enhancement, graph attention neural network, contrastive learning

## Abstract

Aspect-based sentiment analysis (ABSA) is a task of fine-grained sentiment analysis that aims to determine the sentiment of a given target. With the increased prevalence of smart devices and social media, diverse data modalities have become more abundant. This fuels interest in multimodal ABSA (MABSA). However, most existing methods for MABSA prioritize analyzing the relationship between aspect–text and aspect–image, overlooking the semantic gap between text and image representations. Moreover, they neglect the rich information in external knowledge, e.g., image captions. To address these limitations, in this paper, we propose a novel hierarchical framework for MABSA, known as HF-EKCL, which also offers perspectives on sensor development within the context of sentiment analysis. Specifically, we generate captions for images to supplement the textual and visual features. The multi-head cross-attention mechanism and graph attention neural network are utilized to capture the interactions between modalities. This enables the construction of multi-level aspect fusion features that incorporate element-level and structure-level information. Furthermore, for this paper, we integrated modality-based and label-based contrastive learning methods into our framework, making the model learn shared features that are relevant to the sentiment of corresponding words in multimodal data. The results, based on two Twitter datasets, demonstrate the effectiveness of our proposed model.

## 1. Introduction

The internet has revolutionized the way people communicate and share their opinions. With the advent of social media platforms, individuals now have a platform to express their experiences and thoughts on various products, services, and travel destinations. As a result, social media platforms like Twitter and YouTube have become a rich source of user-generated content, including reviews, tags, browser behavior, and shared media objects that convey sentiments and opinions on a wide range of topics. Analyzing this massive amount of user-generated content is essential for potential discoveries [1], affective computing [2], sentiment analysis [3], and behavioral intentions [4]. These techniques can predict human decision-making and enable various applications such as smart sensing, human–computer interaction, and social intelligence. One specific area of sentiment analysis that has garnered significant attention in recent years is aspect-based sentiment analysis (ABSA) [5,6]. This technique is a fine-grained sentiment analysis task that detects sentiment polarities (positive, neutral, or negative) towards specific aspects or entities in the input text.

Various methods have been proposed in the literature for ABSA, like traditional feature-based models [7,8] and deep learning-based models [9,10,11]. In recent years, the trend of fine-tuning pre-trained models in natural language processing (NLP) tasks has led to numerous studies applying the pre-trained BERT model to ABSA [12,13,14]. These approaches have achieved state-of-the-art performance on various benchmark datasets. However, the increasing number of individuals using multimodal content, such as image-text pairs, on social platforms to share their daily experiences or opinions presents a new challenge for sentiment analysis. As a result, recent studies have proposed leveraging useful information from images to enhance the performance of the Multimodal Aspect-Based Sentiment Analysis (MABSA) task. Some studies have proposed new techniques to integrate visual information and text-based information for more accurate MABSA [15,16]. Others are exploring the use of pre-trained models to better understand the relationships between different aspects and entities in a tweet [17,18].

**Figure 1:**
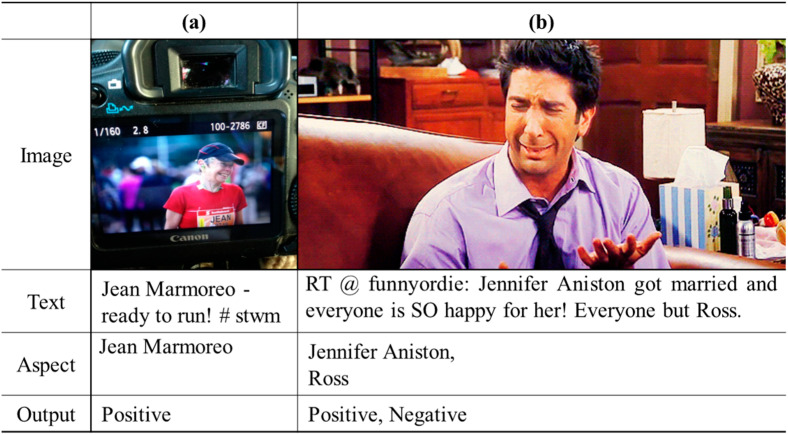
Examples of multimodal aspect-based sentiment analysis. (**a**): one aspect contained in image-text social data. (**b**): two aspects contained in image-text social data.

Despite recent exciting advancements, performing MABSA on social media data such as tweets is a challenging task. This is mainly due to the following reasons: (1) Tweets are often characterized by short text lengths, which lack sufficient information for aspect-based sentiment analysis. Consequently, it becomes difficult to detect the sentiment of a specific aspect without accurately capturing the image content since the critical information for the aspect is often present in the image. For instance, in Figure 1a, the sentiment information for aspect ‘Jean Marmoreo’ is very limited since the text content only says ‘ready to run’ to express the writer’s emotion, which makes it challenging to accurately detect the sentiment of ‘Jean Marmoreo’ without the critical information present in the image. (2) Visual scenes related to tweets are typically hard to relate to aspects in the text, creating a semantic gap between the textual and visual representations. This gap increases the likelihood of misalignment in their inter-modal interactions, which can lead to inaccuracies in aspect-based sentiment analysis. For example, in Figure 1b, the aspect ‘Jennifer Aniston’ only appears in text, and there is a deep correlation between the image and text of the aspect ‘Ross’.

To address the aforementioned limitations, we propose a Hierarchical Fusion Network with Enhanced Knowledge and Contrastive Learning (HF-EKCL) to further improve the performance of aspect-based sentiment analysis by enhancing the knowledge obtained from the image and learning common features related to aspect-based sentiment analysis from different modalities. Specifically, the model consists of three parts: a feature extractor, a hierarchical fusion module, and an aspect-based attention classifier. For the feature extractor, we adopt BERT [19] to extract text, aspect, and caption features. The parameters in BERT are shared when extracting these features, and there is a shallow fusion between text and aspect as well as caption and aspect. The image features are extracted by a pre-trained image encoder. Then, a cross-attention [20] module is applied to exploit element-level interaction between textual and visual features. The structure-level fusion features are obtained by a graph attention neural network [21] using semantic dependencies among words and spatial dependencies among the regions of objects. Finally, an aspect-based attention module is employed to further explore aspect-based sentiment features. To help the model learn the common features related to the sentiment of corresponding words in multimodal data, we add contrastive learning for label and modality. This module leverages the label features among the dataset and enhanced image knowledge of data itself. In this way, the model can learn aspect-based sentiment common features and analyze the sentiment efficiency.

Compared to other aspect-level sentiment analysis models, the main contributions of this study can be summarized as follows:

1. An enhanced knowledge-based hierarchical fusion network is proposed to effectively capture the interactive semantic relationship between different modalities and aspects. The network builds element-level and structure-level fusion features, enhancing the leveraging of multimodal information for aspect-based sentiment analysis.
2. Modality-based and label-based contrastive learning is proposed. By leveraging the label features within the dataset and the enhanced image knowledge extracted from the data itself, the model can learn the common features associated with aspect-based sentiment across different modalities and analyze sentiment efficiency.
3. We conduct extensive experiments and provide thorough ablation studies to demonstrate the effectiveness of our proposed approach to multimodal aspect-based sentiment analysis.

## 2. Related Work

### 2.1. Aspect-Based Sentiment Analysis

Aspect-based sentiment analysis is a field that initially only focused on text, and classical approaches relied on a series of manually designed rules and external knowledge, such as lexical resources, to construct features. Traditional statistical learning methods were then used to predict the sentiment of aspects [22,23]. While these approaches achieved respectable results on various benchmark datasets, they suffered from being labor-intensive and relying heavily on feature engineering. However, recent advancements in deep learning have resulted in a significant number of studies adopting different neural network models to encode the aspect and its related context. For instance, Dong et al. [24] first introduced recurrent neural networks into the aspect-level sentiment classification field. Their approach relied on contextual and syntactic relations to identity sentiment polarities of aspect terms. Liu et al. [25] proposed a new recurrent network that utilized external “memory chains” and a delayed memory update mechanism to better capture linguistic structure. Ma et al. [26] incorporated knowledge from common sense into a hierarchical attention-based deep neural network for aspect-based sentiment classification.

Today, large pretrained language models have become the mainstream building block for aspect-based sentiment analysis. For example, Hoang et al. [27] and Essebbar et al. [28] fine-tuned the pre-trained language model BERT [19] to a sentence pair classification model for the ABSA task in different languages. Other researchers, such as Zhang et al. [29], explicitly modeled the syntactic dependency parsing of the sentence to make predictions by utilizing a Graph Convolutional Network [30] over the sentence’s dependency tree. Chen et al. also proposed a gating mechanism to dynamically combine information from word dependency graphs and latent graphs to improve the performance of the ABSC task [31]. Liang et al. [32] proposed a graph neural network which incorporates the affective information obtained from SenticNet [33] and enhanced the dependency graphs to improve the performance. Nandi et al. [34] introduced the novel concepts of “N-gram Graph-Cut” for aspect-based sentiment analysis and a hybrid approach combining Graph-Cut and LSTM to enhance sentiment classification. Unlike the above methods, our aim in this paper is to expand on this research area by presenting a more effective multimodal approach for the ABSC task.

### 2.2. Multimodal Sentiment Analysis

Existing studies on MSA can be broadly categorized into two types: coarse-grained (sentence-level) and fine-grained (aspect-level) sentiment analysis. For sentence-level sentiment analysis, one of the main challenges is efficiently fusing feature information from different modalities. Early studies attempted to use early or late fusion for sentiment classification, but this had limitations in representing both intra-modality and inter-modality information. To address these limitations, many approaches have explored fine-grained interactions between cross-modalities. For instance, Chen et al. proposed a Gated Multimodal Embedding LSTM with a temporal attention mechanism to better model the multimodal structure [35]. Hazarika et al. designed a new framework that projects modalities into modality-invariant and modality-specific subspaces to achieve a more holistic view of the multimodal data [36]. Paraskevopoulos et al. [37] introduced a neural architecture that adeptly captures cross-modal interactions from a top-down perspective to analyze users’ sentiment. Transformer-based methods have also been proposed for MSA tasks, such as the multi-layer fusion module based on the transformer-encoder developed by Li et al. [38], which incorporates contrastive learning to further explore sentiment features, and the text-enhanced transformer fusion model proposed by Wang et al. to better understand text-oriented pairwise cross-modal mappings and acquire crucial unified multimodal representations [39].

Compared to aspect-based sentiment analysis on plain text, multimodal aspect-based sentiment analysis (MABSA) aims to capture sentiment features from various modal information, improving sentiment expression through joint learning. In recent years, several studies have proposed novel approaches to MABSA and achieved promising results. For instance, Xu et al. [15] developed a co-memory attentional mechanism to better capture interactions between different modalities. Yu et al. [16] proposed an entity-sensitive attention and fusion network that leverages attention mechanisms to capture intra-modality and aggregate features with a textual fusion layer for the MABSA task. To obtain target-sensitive textual/visual representations and achieve multi-modal fusion, Yu et al. [17] developed TomBERT by building upon the baseline BERT architecture and adapting BERT for cross-modal interaction, incorporating self-attention layers. Gu et al. [40] proposed an attention capsule extraction and multi-head fusion network for the task of MABSA, establishing multi-head attention and bidirectional-GRU [41] for textual information while applying the capsule network to handle the fusion features. Khan et al. [42] introduced a two-stream model that first obtains captions for input images and then obtains fusion features through a pretrained BERT model to tackle the task.

## 3. Methodology

### 3.1. Task Definition

For a given multimodal dataset $M=\left\{m_{1},\ldots ,m_{i},\ldots ,m_{k}\right\}$, where $m_{i}$ is a sample from M, and the number of samples is k. Regarding sample $m_{i}$, it consists of a n words sentence $S_{i}=\left\{t_{i}^{1},t_{i}^{2},\ldots ,t_{i}^{n}\right\}$, with l word sub-sequence as aspects $A_{i}=\left\{a_{i}^{1},\ldots ,a_{i}^{j},\ldots ,a_{i}^{l}\right\}$ in $S_{i}$ and an image $\;I_{i}\in {\mathbb{R}}^{3\times h\times w}$, where 3, H, and W represent the number of channels, the height of the image, and the width of the image. Each aspect $a_{i}^{j}$, is assigned a sentiment label $y_{j}\in \;\left\{negative,\;neutral,\;positive\right\}$. Our goal is to develop an aspect-level sentiment classifier that can predict the sentiment $y_{j}\;$ for each aspect $a_{i}^{j}$ by leveraging both the text and image data.

### 3.2. Overview

**Figure 2:**
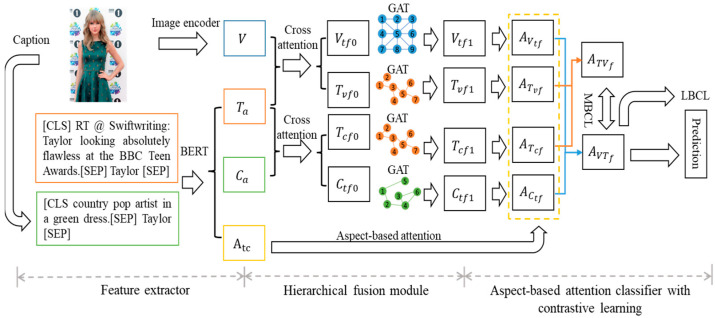
The architecture of the Hierarchical Fusion Network with Enhanced Knowledge and Contrastive Learning (HF-EKCL).

The overall architecture of the proposed Hierarchical Fusion Network with Enhanced Knowledge and Contrastive Learning (HF-EKCL) is as depicted in Figure 2, which consists of three parts: the feature extractor, the hierarchical fusion module, and the aspect-based attention classifier with contrastive learning. Given a multimodal tweet $m_{i}=\left\{S_{i},\;\;I_{i},\;A_{i}\right\}$**,** the following steps are performed: (1) The feature extractor is designed to obtain high-level representations from the image and text data. We use a pre-trained image caption model to generate enhanced knowledge $C_{i}=\left\{c_{i}^{1},c_{i}^{2},\ldots ,c_{i}^{p}\right\}$ for the tweet, where p is the number of caption words. Then, a textual and visual encoder is applied to extract features from different modalities. The text-based aspect representations contain sentence and caption features. (2) Based on the extractor, a hierarchical fusion module focuses on interaction between textual and visual features. We utilize a multi-head cross-attention module for element-level fusion and subsequently implement a graph attention neural network that leverages grammatical dependency trees to establish interconnections among elements, thereby facilitating the extraction of structure-level fusion features. (3) Finally, we apply an attention mechanism with structure-level fusion features to build aspect-based sentiment features to predict the sentiment. In this process, we employ the modality-based contrastive learning (MBCL) and label-based contrastive learning (LBCL) to further learn the aspect-based common sentiment features and improve performance.

### 3.3. Feature Extractor

To generate descriptive captions that contain rich semantic information for each image, we utilize the pre-trained Clipcap [43] model, which has been shown to be highly effective at generating captions that capture the salient aspects of an image while providing a rich description. Similar to [17], we feed $C_{i}$ and aspect $a_{i}^{j}$ into a BERT model using special tokens [CLS] and [SEP] to mark the beginning and separation of the caption and aspect. We then add [SEP] to the end, resulting in $\left[C_{a}:A_{c}\right]$ = BERT([$C_{i}:a_{i}^{j}$]), where $C_{a}\in {\mathbb{R}}^{p\times d}$ and $A_{c}\in {\mathbb{R}}^{b\times d}$ represent the fusion features of caption and aspect. Here, p is the number of caption words, b is the length of the aspect, and d is the hidden dimension. Similarly, we deal with the sentence in the post and aspect using the same pre-trained BERT, $\left[T_{a}:A_{t}\right]$ = BERT([$S_{i}:a_{i}^{j}$]), where $T_{a}\in {\mathbb{R}}^{n\times d}$ represents the aspect-based sentence features and $A_{t}\in {\mathbb{R}}^{b\times d}$ is the sentence-based aspect features, and n is the number of sentence words. The textual fused aspect features $A_{tc}$ contain $A_{c}$ and $A_{t}$; we apply an element-wise sum to these features and use a two-layer MLP to obtain $A_{tc}$, where $A_{tc}\in {\mathbb{R}}^{b\times d}$. For image $\;I_{i}$, we first resize it to 224 × 224 and divide the image into r patches. These patches are then reshaped into a sequence and are fed into the pre-trained Vision Transformer [44] to build visual features V, where $V\in {\mathbb{R}}^{r\times d}$. The textual features and visual features have the same hidden dimension.

### 3.4. Hierarchical Fusion Module

#### 3.4.1. Element-Level Fusion

To build element-level (words-level/region-level) interaction between visual and textual features, we use a cross-attention mechanism with h heads to firstly align different modalities to reduce the gap between image and text. The i-th head cross-attention for sentence features $T_{a}$ and visual features V is defined as:

(1) headi=SoftmaxTaWqiTdhVWkiVWvi,

where $W_{q}^{i}\in {\mathbb{R}}^{d\times \frac{d}{h}}$, $W_{k}^{i}\in {\mathbb{R}}^{d\times \frac{d}{h}}$ and $W_{v}^{i}\in {\mathbb{R}}^{d\times \frac{d}{h}}$, are trainable query, key and value projection parameter matrices and $head_{i}\in {\mathbb{R}}^{n\times \frac{d}{h}}$. Then, we concatenate the heads features as the first layer fused features, which is the input for second layer attention. Several cross-attention layers are applied to complete the fusion. We finally employ two-layer MLP with residual connection to obtain visual region-aware aspect-based sentence features $T_{vf0}$:

(2) $$T_{vf0}=LN\left(T_{a}+MLP\left(\left[head_{1}:head_{2}:\ldots :head_{h}\right]\right)\right),$$

where $T_{vf0}\in {\mathbb{R}}^{n\times d}$, *LN* represent the layer normalization function and “:” donate the concatenate operation. Similar to $T_{vf0}$, we utilize the same structure to obtain sentence word-aware image features $V_{tf0}\in {\mathbb{R}}^{r\times d}$, caption word-aware sentence features $T_{cf0}\in {\mathbb{R}}^{n\times d}$, and sentence word-aware caption features $C_{tf0}\in {\mathbb{R}}^{p\times d}$.

#### 3.4.2. Structure-Level Fusion

**Figure 3:**
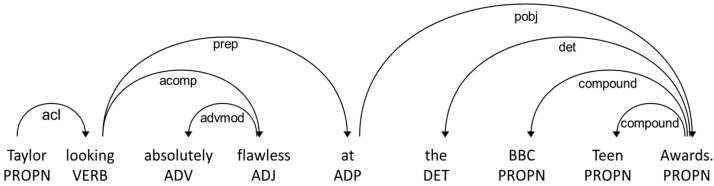
An example of a dependency tree generated by Spacy.

Structure-level fusion aims to deal with the complicated structures that naturally exist in visual and textual modalities. To achieve this, we build a textual and visual graph that encompasses element-level fused features. This construction is rooted in the dependency relations that exist among words and the location relations of visual features. For the extraction of syntactic relationships among words, we employ an open-source natural language processing (NLP) library named spaCy (https://spacy.io/, accessed on 14 January 2023). This library provides a comprehensive set of tools to analyze the syntactic structure of sentences and extract the grammatical relationships between words. We utilize the dependency parser provided by spaCy to extract the edges for the dependency graph. This parser identifies the grammatical relationships between words, such as subject verb–object and noun–modifier, and represents them as directed edges in the dependency tree. Figure 3 illustrates this with an example of interconnected words, and in the textual graph, the aspect “Taylor” and the verb “looking” are connected, indicated by a distance of 1, denoting their grammatical correlation. In the absence of a direct link, the distance remains 0.

Building the visual graph involves beginning with r image patches, with each patch being treated as an individual node within the graph. These nodes are then connected based on their geometric closeness. In particular, each node is directly linked to its 8 neighboring nodes, which effectively captures the spatial relationships existing among the image patches. That is, an edge with a value of 1 signifies a direct connection between a node and its neighboring nodes, while an edge with a value of 0 indicates no direct connection to other nodes. Note that both types of graphs, which are created from a grammar dependency tree and based on geometrical adjacency, respectively, are undirected. Then, the graph attention network (GAT) [21] is designed to build structure-level fusion; it uses self-attention layers to determine the importance of information flowing between nodes. By employing GAT, we can propagate semantic information at the element level along with the graph edges, allowing us to learn comprehensive representations at the structure level for both textual and image modalities. Considering the aspect itself does not express the emotion, the self-loop is not applied for the structure-level fusion network. The graph attentional weight for $T_{vf0}{}_{i}$ and $T_{vf0}{}_{j}$, from the visual-aware sentence features $T_{vf0}$, is illustrated as:

(3) $$\alpha_{ij}=\frac{\exp\left(LKReLU\left(e^{T}\left[W_{l}T_{vf0}{}_{i}:W_{l}T_{vf0}{}_{j}\right]\right)\right)}{\sum_{k\in N_{i}}\exp\left(LKReLU\left(e^{T}\left[W_{l}T_{vf0}{}_{i}:W_{l}T_{vf0}{}_{k}\right]\right)\right)},$$

where LKReLU represents the LeakyReLU activation function; $e\in {\mathbb{R}}^{2d}\;$ and $W_{l}\in {\mathbb{R}}^{d\times d}$ are the learnable weight parameters of GAT. $k\in N_{i}$ is the neighborhood of $T_{vf0}{}_{i}$ in the dependency graph. $\alpha_{ij}$ indicates the importance of node j’s features to node i. To stabilize this learning process, multi-head attention is applied; the attention weights that have h attention head are $\alpha_{ij}=\left\{\alpha_{ij}{}_{1},\alpha_{ij}{}_{2},\;\ldots ,\alpha_{ij}{}_{h}\right\}$; therefore, for $T_{vf0}{}_{i}$, the first GAT-layer output structure-level feature $T_{vf0}{}_{i}^{1}$ is represented as

(4) $$T_{vf0}{}_{i}^{1}=\frac{1}{h}\overset{h}{\underset{r=1}{\sum }}\underset{k\in N_{i}}{\sum }\alpha_{ij}{}_{r}W_{l}T_{vf0}{}_{k}.\;$$

We can obtain the features $T_{vf0}{}_{i}^{l}$ from the output of the l−th GAT layer in a similar manner. Additionally, we generate the structure-level-fused sentence features $T_{vf0}$ from the l-th GAT layer, which consists of n tokens. The results are represented as $T_{vf1}=\left\{T_{vf0}{}_{1}^{l},\;T_{vf0}{}_{2}^{l},\ldots ,T_{vf0}{}_{n}^{l}\right\}$ and $T_{vf1}\in {\mathbb{R}}^{n\times d}$. Likewise, we can obtain other structure-level representations, such as $V_{tf1},\;T_{cf1},\;C_{tf1}$.

### 3.5. Hierarchical Fusion Module

#### 3.5.1. Aspect-Based Attention Module

An aspect-based attention module is employed to learn deep interactions between aspects and fused features. Firstly, we use a multi-head self-attention mechanism, which is similar to the multi-head cross-attention mentioned in Section 3.4.1, and one-dimensional max pooling to extract important information from fused features for aspect $A_{tc}$ denoted as $\hat{A_{tc}}$. The calculation of $\hat{A_{tc}}$ is as follows:

(5) $$\hat{A_{tc}}={\left(Maxpooling\left(SelfAtt{\left(A_{tc}\right)}^{T}\right)\right)}^{T},$$

$\hat{A_{tc}}\in {\mathbb{R}}^{1\times d}$ is a representative feature that can be used to build a correlation with the fusion features. We consider $c=ReLU\left(\hat{A_{tc}}W_{a}\right)$ and $W_{a}\in {\mathbb{R}}^{d\times d}$ to be the learnable parameters and c $\in {\mathbb{R}}^{1\times d}$. The feature c can be used to construct reliable features from fused aspect features, addressing the issue of insufficient words in a sentence. We then concatenate c with textual-orientated-fused features $T_{vf1}$ and $T_{cf1}$ as the target features for attention. For the aspect-based sentence-fused attention feature $A_{T_{vf},}\in {\mathbb{R}}^{d}$ is computed as follows:

(6) $$A_{T_{vf}}=sum\left(\left[T_{vf1}:c\right]⊙Softmax\left(\frac{\left[T_{vf1}:c\right]{\hat{A_{tc}}}^{T}}{\sqrt{n}}\right)\right),$$

where ⊙ is an element-wise vector product. The aspect-based attention fused features, $A_{T_{cf}},$ can be calculated in a similar way. To obtain the visual aspect-based attention features, $A_{V_{tf}}\;\mathrm{and}\;A_{C_{tf}}$, we directly use the structure-level fusion features and $\hat{A_{tc}}$ for calculation. Using $A_{V_{tf}}$ as an example, the formula is as follows:

(7) $$A_{V_{tf}}=sum\left(V_{tf1}⊙Softmax\left(\frac{V_{tf1}{\hat{A_{tc}}}^{T}}{\sqrt{n}}\right)\right).\;$$

Finally, we concatenate the visual-orientated features $A_{V_{tf}}$ and $A_{C_{tf}}$ to build textual aspect sentiment features $A_{VT_{f}}\in {\mathbb{R}}^{2d}$ and apply two-layer MLP and Softmax activation function to predict the sentiment of aspect $y^{\prime }$*:*

(8) $$y^{\prime }=Softmax(MLP\left(A_{V_{tf}}:A_{C_{tf}}\right).\;$$

#### 3.5.2. Contrastive Learning

Similar to the authors of [38], we divided contrastive learning into two parts: modality-based contrastive learning (MBCL) and label-based contrastive learning (LBCL). Modality-based contrastive learning enhances the consistency of sentiment analysis between text and visual modalities, thereby helping the model explore effective sentiment features between images and sentences. Since aspect-based sentiment features should exist in either image or sentence features, MBCL enables the model to find features related to sentiment in both images and sentences and align the sentiment features between different modalities. The batch size image-orientated aspect sentiment features $A_{VT_{f}}{}_{BS}\in {\mathbb{R}}^{BS\times 2d}$ are a stack of $A_{VT_{f}}$, and the sentence-orientated aspect sentiment features $A_{TV_{f}}{}_{BS}\in {\mathbb{R}}^{BS\times 2d}$ are obtained by concatenating batch size $A_{T_{vf}}{}_{BS}$ and $A_{T_{cf}}{}_{BS}$. The loss function for batch size MBCL is shown in Equation (9):

(9) $$loss_{MBCL}=CE\left(\frac{MLP_{T}\left(A_{TV_{f}}{}_{BS}\right)MLP_{V}{\left(A_{VT_{f}}{}_{BS}\right)}^{T}}{\tau },\;ag\left(BS\right)\right),$$

where BS represents batch size, τ is contrastive learning’s temperature, and CE is the cross-entropy function; ag is the arange function. LBCL is also applied to learn the sentiment-related features among multimodal data. For each batch size data, we separate them into 3 groups (positive, negative, and neutral) according to their labels; the same label group samples (like positive and positive samples), are regarded as positive samples for contrastive learning, the different label two groups are negative samples for contrastive learning (like positive-negative, positive neural samples). In the batch, samples that have the same neutral label are regarded as positive samples for learning, and the samples with positive or negative labels are considered negative labels for learning. The specific algorithm for LBCL is as follows:

(10) $$loss_{LBCL}=\frac{gather\left(LSF\left(\frac{MLP_{T}\left(A_{VT_{f}}{}_{BS}\right)MLP_{T}{\left(A_{VT_{f}}{}_{BS}\right)}^{T}}{\tau }\right),M_{ind}=1\right)}{BS},$$

where $M_{ind}\in {\mathbb{R}}^{BS\times BS}$ is the index matrix, and $M_{ind}{}_{i,j}=1$ when sample i  and sample j have the same sentiment label; otherwise, $M_{ind}{}_{i,j}=0$. gather means gathers the values when its corresponding index matrix value is 1, and LSF represents the LogSoftmax function. τ is the contrastive learning temperature, similar to MBCL.

### 3.6. Final Objective Function

The final objective of the model is to minimize the loss function in order to optimize all of its parameters. In the case of aspect-based sentiment classification, the categorical cross-entropy is used as the loss function. Therefore, the total loss function for the model that combines contrastive learning can be expressed as follows:

(11) $$L_{total}=CE\left(y^{\prime },y\right)+\lambda_{M}loss_{MBCL}+\lambda_{L}loss_{LBCL},$$

where $y^{\prime }$ is the prediction result of sentiment, and y is the corresponding label; to balance the different training losses, $\lambda_{M}$ and $\lambda_{L}$ are coefficients.

## 4. Experiments

### 4.1. Dataset and Model Settings

To assess the effectiveness of our research questions, we utilize two benchmark datasets for aspect-based multimodal sentiment classification: Twitter-15 and Twitter-17. These datasets were introduced by the authors of [17], and these datasets consist of multimodal user posts that were published on Twitter between 2014 and 2015 and 2016 and 2017, respectively. Each dataset consists of multimodal tweets that include text, images, targets within the tweet, and the sentiment of each target. The sentiment of each aspect is labeled as positive, neutral, or negative from a set of three possible labels so the task involves standard multi-class classification. Table 1 presents an overview of the basic information for the two datasets, specifically focusing on the amount of data available for each dataset (Twitter-15 and Twitter-17), the average aspect, and the length of sentences in the dataset. It is worth noting that the distribution of labels in the training, validation, and test sets is similar. Furthermore, the average sentiment distribution across sentences remains consistent in both datasets. This consistency indicates that the overall sentiment tendencies are comparable. We trained our model using various hyperparameters, and their respective values are provided in Table 2. During the training process, we utilized the Adam optimizer [45] to schedule the learning rate. The PyTorch framework facilitated the implementation of our model, and we evaluated the model’s performance using accuracy and Macro-F1 metrics (as in previous studies).

### 4.2. Baselines

To evaluate the performance of the proposed model (HF-EKCL), we conducted a comparative analysis with several existing models encompassing various approaches, including visual sentiment analysis methods, textual sentiment approaches, and multimodal sentiment models.

1. ResNet-Aspect: Utilizes visual features and aspect embeddings, which are extracted using ResNet and BERT, respectively. An attention layer is then applied to integrate all these features and embeddings and predict aspect-based sentiment analysis.
2. ATAE [46]: Aspect embeddings are added to the attention-based Long Short-Term Memory (LSTM) networks, allowing the model to better capture important contextual information related to the aspect.
3. RAM [47]: Employs position-based weighting and multiple attention mechanisms to construct attention-based features. These features are then processed using a non-linear combination with GRU to predict sentiment for targets.
4. MGAN [48]: Combines a fine-grained attention mechanism with a coarse-grained attention mechanism to capture word-level interactions between aspects and context, along with an aspect alignment loss to capture aspect-level interactions for the analysis.
5. BERT [19]: A pre-trained language model that uses a stacked Transformer encoder architecture to capture bidirectional context, generate context-aware word features, and explore the relationship between the aspect and the sentence.
6. MIMN [15]: Proposes a multi-interactive memory network for aspect-based sentiment analysis that uses two memory networks to model text and image data; contains multiple memory hops for attention extraction.
7. ESAFN [16]: Utilizes attention mechanisms to generate aspect-sensitive textual representations and aspect-sensitive visual representations using an oriented visual attention mechanism. These are then fused with a bilinear interaction layer for prediction.
8. EF-Net [40]: TABMSA uses an attention capsule extraction and multi-head fusion network with multi-head attention and ResNet-152 to analyze the sentiment of targeted aspects in a multimodal setting.
9. ViBERT [49]: An extension of the BERT model that includes multiple pre-trained Transformer layers applied to the concatenation of both text and image features extracted from BERT and Faster R-CNN, respectively.
10. Tombert [17]: Uses a target attention mechanism to derive aspect-sensitive visual representations by performing aspect–image matching and stack self-attention layers to capture multimodal interactions.
11. EF-CapTrBERT [42]: Employs image translation in the input space to convert images into text. The resulting text is then combined with an auxiliary sentence and fed into the encoder of a language model using multimodal fusion.

### 4.3. Main Results

Table 3 provides a detailed comparison between the proposed HF-EKCL model and various baseline models on the Twitter-15 and Twitter-17 datasets. It is evident that our proposed model achieves the best results for the Twitter-15 and Twitter-17 datasets. Based on these results, we can draw the following conclusions: (1) The ResNet-Aspect model’s performance is limited, and its accuracy results (around 60%) highlight the importance of textual information. (2) The ATAE model performs the worst in classical ABSA tasks as it only concatenates the representations of sentences and aspects, losing the effective correlation between text and aspect. On the other hand, RAM and MGAN improve sentiment analysis results by utilizing the designed interaction module between aspect and text. BERT, with its pre-trained parameters and deep architecture, achieved the best results in ABSA tasks. (3) Unimodal baseline approaches that do not use transformers to obtain features generally perform worse than multimodal approaches. This suggests that combining image and text information can lead to improved sentiment classification performance. (4) Compared to BERT, the ViLBERT model does not explicitly model the interactions between aspect–text and aspect–image, which worsens performance. In contrast, the EF-CapTrBERT model, which incorporates enhanced knowledge and a transformer architecture, outperforms other multimodal approaches. (5) The overall performance of HF-EKCL is better than that of EF-CapTrBERT, indicating the effectiveness of the proposed hierarchical fusion architecture with enhanced knowledge and contrastive learning. Our proposed model outperforms EF-CapTrBERT, demonstrating a significant improvement in Macro-F1 on both open datasets. Specifically, we can observe a 2.14% and 1.46% increase in Macro-F1 for the two open datasets, respectively. Our model also achieved an accuracy that was 1.6% higher when evaluated on the Twitter 2017 dataset.

### 4.4. Ablation Studies

To further investigate the impact of different modules in the proposed HF-EKCL model, we conducted ablation experiments by constructing several variants. In Table 4, we present the results obtained by removing contrastive learning, enhanced knowledge of the image, and the structure-level fusion layer, respectively. Our observations reveal that all the modules contributing to HF-EKCL are essential for the model’s performance, with the structure-level fusion layer being the most crucial module, as demonstrated by the notably lower results compared to the others. Furthermore, we found that the model’s performance without contrastive learning is significantly worse than that of the complete model, highlighting the effectiveness of learning common features between modalities and labels. Moreover, the moderate performance drop observed after removing enhanced knowledge of the image underscores the importance of this module.

**Figure 4:**
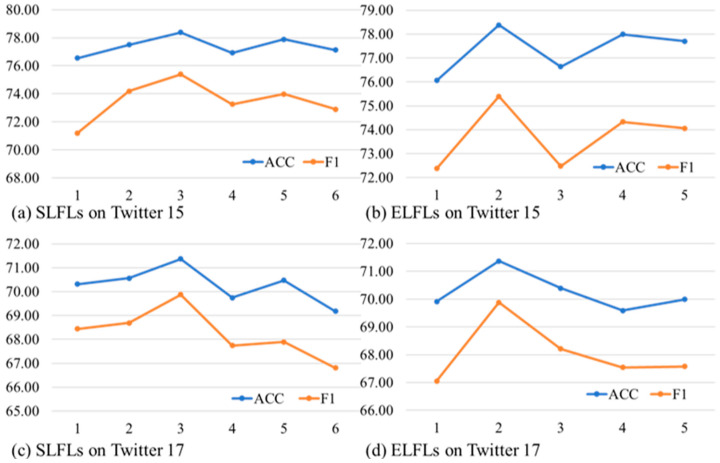
The results of different element-level fusion layers (ELFL) and structure-level fusion layers (SLFL) on two datasets. The x-axis represents the number of layers.

We measured the change in performance by varying the number of element-level fusion layers (ELFL) and structure-level fusion layers (SLFL) on the Twitter-15 and Twitter-17 datasets. The number of ELFL layers ranged from 1 to 6, while the number of SLFL layers ranged from 1 to 5, and the results are illustrated in Figure 4. By comparing the results shown in Figure 4a,c, we can see that the results pertaining to the performance of different ELFL on both datasets are similar. We can observe that the best accuracy and F1 score is achieved when the number of layers is three, which suggests that excessive cross-attention layers may mismatch the element alignment between image and text. In terms of the structure-level fusion layers, we can observe that the best results are achieved using a two-layer GAT model. The performance of the structure-level fusion layers decreases rapidly after two layers, with only minor improvements being observed afterward. We speculate that increasing the layer number may lead to the problem of nodes becoming indistinguishable from each other, which could be a contributing factor to the decrease in performance.

### 4.5. Case Study

**Figure 5:**
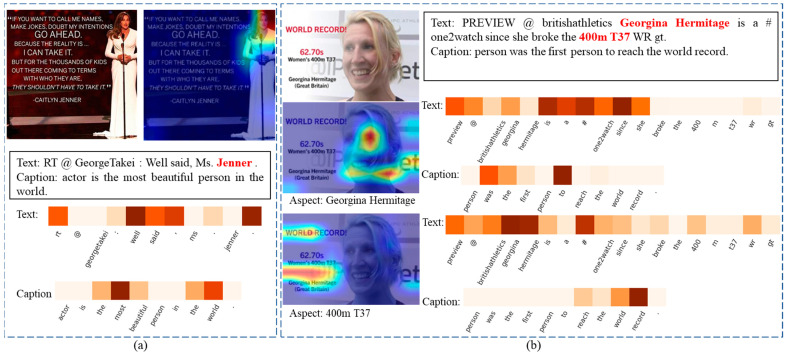
Examples of attention weights for a sentence and an image for different aspects. (**a**): The attention heat map for one aspect image-text pair. (**b**): The attention heat map for two aspects image-text pair.

We visualized the attention areas for images and text for a single aspect in a sentence and for two aspects in a post. We present the attention visualization results in Figure 5a,b, where the red color indicates importance for images, and darker shades indicate importance for sentiment analysis in the text. We observed that, for sentiment prediction, the model focuses on the character that appears in the image and the key information in the text, such as the words ‘well’ and ‘most’. Although there are two aspects in the post, the model predicts the results correctly for both, and the important areas differ for each aspect. When the aspect is ‘Georgina Hermitage’, the model focuses on the person appearing in the image and pays more attention to the actions and descriptions related to that person in the text content. On the other hand, when the aspect is ‘499m T37,’ the model focuses on the background of the image, which is relevant to ‘400m T37′, and identifies the words that most closely correlate to the aspect in the sentence and caption for sentiment analysis. These attention weight visualization results indicate that the model accurately finds the correlation between aspect–image and aspect–text and adapts its attention accordingly.

## 5. Conclusions

In this paper, we addressed the task of aspect-based sentiment classification with multimodal data, specifically image and sentence inputs. We proposed a hierarchical fusion network that leverages enhanced knowledge and contrastive learning (HF-EKCL) for sentiment analysis. Our approach utilizes a multi-level fusion architecture to effectively capture the interaction between image and sentence, while enhanced knowledge is employed to better understand the content of image. Additionally, we designed modality-based and label-based contrastive learning mechanisms to improve the model’s ability to extract common sentiment-related features from the data. The experimental results derived from testing on two public datasets demonstrate the effectiveness of our proposed approach, and the visualizations reveal the model’s ability to observe the interaction between aspects and their corresponding content for sentiment classification.

## 6. Limitations and Further Work

Despite the improved performance of the model, the method does have its limitations. The utilization of Twitter data from older datasets in this study led to constraints in analyzing current Twitter trends. Moreover, the experimental data underwent artificial interventions, deviating from the authenticity of real-world multimedia data. Therefore, the generalization ability of the model may be reduced. Our future work will be concentrated on the field of end-to-end aspect-based real-time multi-modal sentiment analysis. This approach has the potential to serve as a valuable sentiment sensor for opinion polls. By focusing on real-time dynamics, it addresses some existing limitations and provides stronger and more accurate sentiment analysis in a changing public opinion environment.

## Figures and Tables

**Table 1:** The statistics of multimodal aspect-based sentiment analysis.

Split	Twitter-15						Twitter-17					
	Pos	Neu	Neg	Total	AvgAspect	Len	Pos	Neu	Neg	Total	AvgAspect	Len
Train	928	1883	368	3179	1.34	16.72	1508	1638	412	3562	1.41	16.21
Valid	303	670	149	1122	1.33	16.74	512	517	144	1176	1.43	16.37
Test	317	607	113	1037	1.35	17.05	493	573	168	1234	1.45	16.38

**Table 2:** The hyperparameter settings of the model.

Parameters	Value
Max length of sentence	50
Max length of image caption	50
Embedding dimension	400
Layer number for Element-level fusion	3
Head number for cross-attention	5
Layer number for Structure-level fusion	2
Head number for graph attention	2
Weight for MBCL, $\lambda_{M}$	1
Weight for LBCL, $\lambda_{L}$	1
Batch size	16
Learning rate	2 × 10^−5^
Weight decay	5 × 10^−3^
Dropout rate	0.5
Max length of sentence	50
Max length of image caption	50
Embedding dimension	400

**Table 3:** Performance of baseline models and the proposed model.

Modality	Method	Twitter-15		Twitter-17	
		ACC	F1	ACC	F1
Image	ResNet-Aspect	59.49	47.79	57.86	53.98
Text	ATAE	70.30	63.43	61.67	57.97
	RAM	70.68	63.05	64.42	61.01
	MGAN	71.17	64.21	64.75	61.46
	BERT	74.15	68.86	68.15	65.23
Image + text	MIMN	71.84	65.59	65.88	62.99
	ESAFN	73.38	67.37	67.83	64.22
	EF-Net	73.65	67.9	67.77	65.32
	ViBERT	73.76	69.85	67.42	64.87
	TomBERT	77.15	71.75	70.34	68.03
	EF-CapTrBERT	78.03	73.25	69.77	68.42
	HF-EKCL (ours)	78.38	75.39	71.37	69.88

**Table 4:** Results from the ablation study of HF-EKCL.

Method	Twitter-15		Twitter-17	
	ACC	F1	ACC	F1
w/o Contrastive learning	76.83	73.09	70.48	68.65
w/o enhanced knowledge	77.90	73.93	71.05	69.03
w/o structure-level fusion	76.83	72.08	69.42	67.80
HF-EKCL	78.38	75.39	71.37	69.88

## Data Availability

The data presented in this study belong to public datasets.
